# Resistance profile for pathogens causing urinary tract infection in a pediatric population, and antibiotic treatment response at a University Hospital, 2010-2011

**Published:** 2014-03-30

**Authors:** Catalina Vélez Echeverri, Lina María Serna-Higuita, Ana Katherina Serrano, Carolina Ochoa-García, Luisa Rojas Rosas, Ana María Bedoya, Margarita Suárez, Catalina Hincapié, Adriana Henao, Diana Ortiz, Juan José Vanegas, John Jairo Zuleta, David Espinal

**Affiliations:** 1 Pediatric Nephrologist, Hospital Pablo Tobón Uribe. Medellín Colombia; 2. Pediatric Nephrologist, Universidad de Antioquia. Medellín Colombia; 3 Medical Laboratory Technician, Hospital Pablo Tobón Uribe. Medellín Colombia; 4. Clinical Epidemiologist, Hospital Pablo Tobón Uribe. Medellín Colombia; 5 Pediatrician, Hospital Pablo Tobón Uribe. Medellín Colombia

**Keywords:** Urinary tract infection, drug resistance, bacterial, Escherichia coli

## Abstract

**Introduction:**

Urinary tract infection (UTI) is one of the most common bacterial infections in childhood and causes acute and chronic morbidity and long-term hypertension and chronic kidney disease.

****Objectives**:**

To describe the demographic characteristics, infectious agents, patterns of antibiotic resistance, etiologic agent and profile of susceptibility and response to empirical treatment of UTI in a pediatric population.

**Methods:**

This is a descriptive, retrospective study.

**Results:**

Included in the study were 144 patients, 1:2.06 male to female ratio. The most common symptom was fever (79.9%) and 31.3% had a history of previous UTI. 72.0% of the patients had positive urine leukocyte count (>5 per field), urine gram was positive in 85.0% of samples and gram negative bacilli accounted for 77.8% for the total pathogens isolated. The most frequent uropathogens isolated were *Escherichia coli and Klebsiella pneumoniae. *Our *E.coli* isolates had a susceptibility rate higher than 90% to most of the antibiotics used, but a resistance rate of 42.6% to TMP SMX and 45.5% to ampicillin sulbactam. 6.3% of *E. coli *was extended-spectrum beta-lactamases producer strains. The most frequent empirical antibiotic used was amikacin, which was used in 66.0% of the patients. 17 of 90 patients who underwent voiding cistouretrography (VCUG) had vesicoureteral reflux.

**Conclusion:**

This study revealed that *E. coli* was the most frequent pathogen of community acquired UTI. We found that *E. coli* and other uropathogens had a high resistance rate against TMP SMX and ampicillin sulbactam. In order to ensure a successful empirical treatment, protocols should be based on local epidemiology and susceptibility rates.

## Abstract (es)

**Antecedentes:**

La infección del tracto urinario (ITU) es una de las infecciones bacterianas más frecuentes en la infancia, se asocia con alta morbilidad aguda y de enfermedades a largo plazo como hipertensión arterial e insuficiencia renal crónica.

**Objetivo:**

Describir las características demográficas, agente etiológico, perfil de sensibilidad y respuesta al tratamiento empírico de una población pediátrica.

**Métodos:**

Estudio descriptivo, retrospectivo realizado en el Hospital Pablo Tobón Uribe (HPTU), Medellín (Colombia), entre enero de 2010 y diciembre de 2011.

**Resultados:**

Se incluyeron 144 pacientes, con una relación hombre: mujer de 1:2.06. El 79.9% consultó por fiebre objetiva y el 31.3% había tenido un episodio de ITU previa. El 72.0% presentó leucocituria y el gram de gota de orina sin centrifugar fue positivo en el 86.0% (77.8% de bacilos con tinción de gram negativos). Los uropatógenos más frecuentes fueron *Escherichia coli y Klebsiella pneumoniae*. *Escherichia coli *tuvo una sensibilidad mayor al 90% para la mayoría de antibióticos, excepto en el de trimetropín sulfametoxazol, con una resistencia del 42.6% y del 45.5% a la ampicilina sulbactam. Se usó la amikacina como tratamiento empírico en el 66.0%. En 17 de 90 pacientes se encontró algún grado de reflujo vesicoureteral (RVU).

**Conclusión:**

La elección del tratamiento empírico precoz debe basarse en el conocimiento de la epidemiología y el perfil de sensibilidad y resistencia de cada institución. En este estudio se encontró elevado porcentaje de resistencia de la *E. coli* y otros uropatógenos a trimetropín sulfametoxazol y ampicilina sulbactam.

## Introduction

The urinary tract infection (UTI) is one of the most common bacterial infections of childhood 1 and it is associated with significant acute morbidity and long-term illnesses such as arterial hypertension 2 and chronic renal failure 3 ^,^ 4 which is why it is necessary to make an early diagnosis, provide effective treatment and appropriate follow-up.

The epidemiology of UTI varies according to age and sex. About 5% of girls and 2% of boys experience at least one episode of urinary tract infection 5. The global prevalence in children under two years of age is 7% 6. The presentation of UTI can be limited with compromise of the lower urinary tract or may be extend to the renal parenchyma and produce a systemic inflammatory response 1. In infants, due to their inability to identify or communicate their symptoms, a high degree of suspiciousness by the physician is required to make an early diagnosis in order to initiate appropriate treatment and avoid later complications, such as renal scarring, hypertension and chronic renal disease 7.

Suspecting UTI, the start of empirical therapy is indicated, especially in younger patients. This should ideally be supported by local epidemiological services at each institution to increase the likelihood of therapeutic success. There is now a greater concern with the increased likelihood of antimicrobial resistance which is reflected in a greater number of treatment failures with drugs that have previously been considered frontline. To reduce the rate of resistance it is important to redirect antibiotic treatment after microbiological confirmation and determination of its sensitivity 8.

To elucidate studies that allow determination of the local epidemiology of frequent uropathogens in UTI and their antimicrobial resistance encourages higher cure rates and a more rational use of antibiotics. This study has the objective of describing the demographic characteristics, etiologic agent, resistance profile and response to empirical treatment for a pediatric population cared for at a medical institution in Medellin that provides for diagnosis UTI during the years 2010 and 2011.

## Material and Methods

This is a cross-sectional study that was conducted at the Pablo Tobon Uribe Hospital (HPTU), a level-four university hospital located in the city of Medellin, Colombia. This study evaluated positive urine cultures processed in the microbiology laboratory of HPTU taken from pediatric patients (ages 0-14 years) who were seen in the emergency department or outpatient clinic for suspected UTI during the period between January 2010 and December 2011. The diagnosis of UTI was made by means of a positive urine culture for one organism with a count greater than or equal to 50,000 colony forming units (CFU) if it was collected by a urinary evacuating catheter and greater than or equal to 100,000 UFC, if it was collected by spontaneous voiding, according to criteria established by the American Pediatric Academy 9. The choice of sampling (evacuating bladder catheter vs. spontaneous voiding) was done in accordance with accepted guidelines for each age group.

Patients with malnutrition, primary immunodeficiency, lymphoproliferative disease, liver cirrhosis, chronic renal disease, and neurogenic bladder and patients treated with steroids and chemotherapy were excluded from the study. Also excluded were patients in which a urine culture was first taken 24 hours after admission to the emergency room.

From the database of the microbiology laboratory a number of histories were obtained for patients who met the inclusion criteria for the study period. Clinical and demographic information was obtained from electronic medical records. Demographic data were collected including age in months at time of diagnosis, sex, clinical diagnoses, results of urine cultures and antibiogram, full blood count, C-reactive protein (CRP) and urinalysis. Also included were data on utilized empirical treatments (previously used antibiotics before knowing microbiological isolations and sensitivity profile), antibiotic treatment time in days, clinical response (resolution of fever and other clinical improvements in the symptoms reported at the time of diagnosis) and complications associated with the infection, such as abscess, nephronia or pyelonephritis.

The data were entered in a previously designed form and were analyzed with SPSS 17.0 (SPSS Inc., Chicago, IL, USA). A descriptive analysis of the data was performed, including calculations of frequencies and proportions for the qualitative variables and quantitative variables were described in terms of means or medians with their respective standard deviations or percentiles according to the distribution of the data identified by the Shapiro Wilkerson test. To assess association between qualitative variables chi-square tests were run with a significance level of 0.05. This study was approved by the Ethics Committee of the Pablo Tobon Uribe Hospital. It followed the rules on ethical aspects of research on human subjects contained in Resolution 008430 of 1993 from the Ministry of Health in Colombia and the confidentiality of the data from patients included in the study was honored.

## Results

### Demographic characteristics:

One hundred forty-four (144) patients were included with a median age of 28 months (p25-75: 13-48). The minimum age at diagnosis ranged from 0 months to a maximum of 180 months. Demographic characteristics are recorded in Table 1. The ratio of male to female was 1:2, however, the age group ratios were found to be: 1:1 in children under 1 year of age, 1:1.7 in those 1-2 years old, 1:4 in those 2 to 5 years of age and 1:1.5 for those older than 5 years.

**Table 1:**
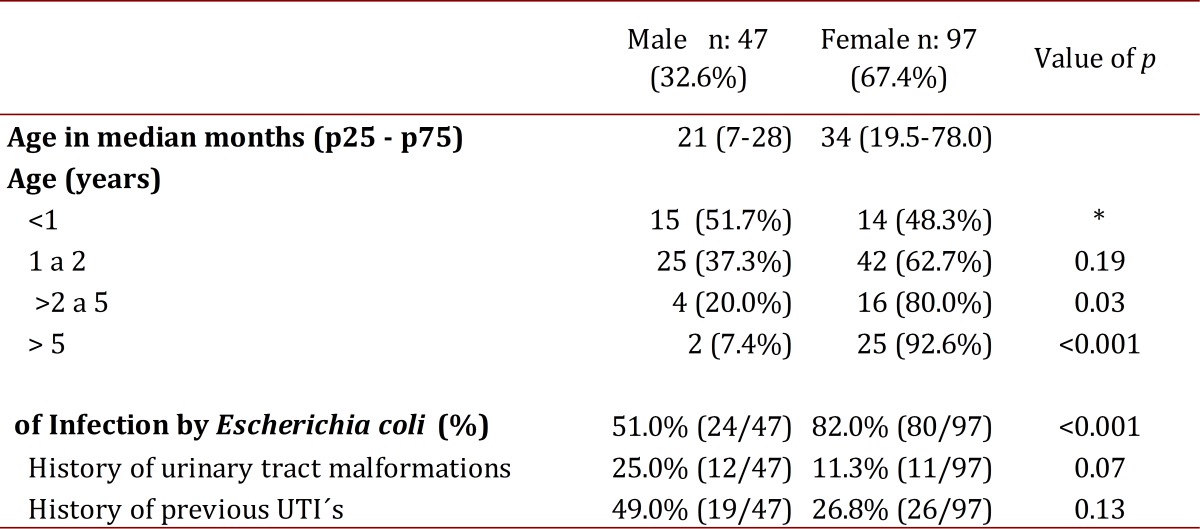
Demographic Characteristics.

### Clinical results:

Reported symptoms at diagnosis were: fever quantified by thermometer (79.9%), vomiting (32.6%), irritability (28.5%), abdominal pain (28.5%), dysuria (20.8%), diarrhea (15.0%), seizures (10.4%) and positive fist percussion was reported in 6.9% of patients.

Fever was the most frequent clinical finding for all ages. Vomiting, abdominal pain and dysuria predominated in those over two years of age, and in children under two years of age irritability, diarrhea and vomiting were predominant (Table 2). Respiratory symptoms were found in 27.8% of all patients, but they were more common in children under 2 years of age (47.8%).

**Table 2:**
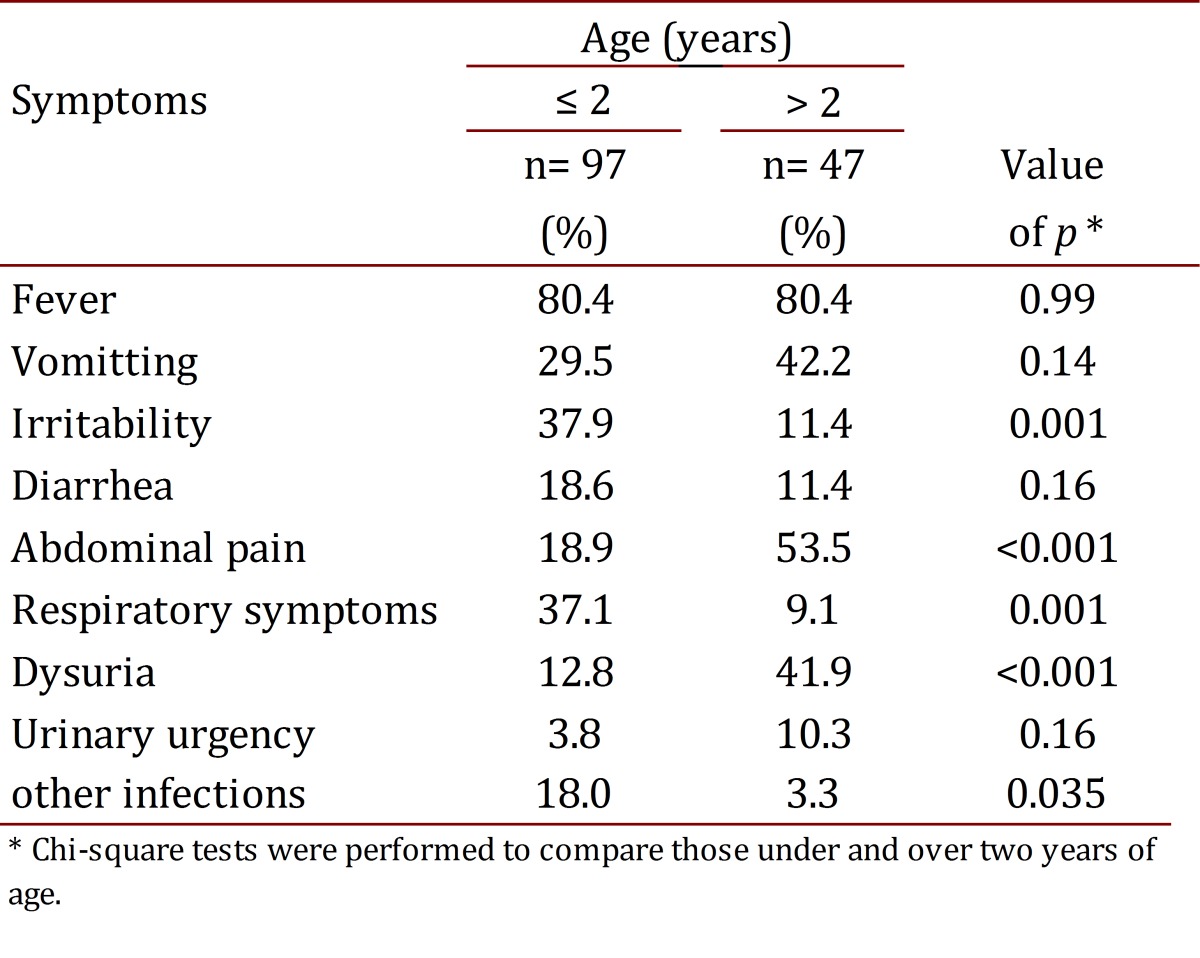
Presence of symptoms according to age group

### Risk factors for UTI:

Among the risk factors described in the literature associated with UTI, it was found that 31.3% of patients had a prior episode of UTI, and 14.6% had more than one previous episode of UTI. Other reported factors were constipation (6.3%), vesicoureteral reflux (VUR) (14.6%) and previous renal scarring (6.9%).

### Laboratory characteristics:

Seventy-four point four percent (74.4%) (96/129) of patients had a high CRP, with a median of 4.19 mg/dL (p25-75: 0.95-8.29), with a minimum of 0.01 mg/dL and maximum of 37 mg/dL at the time of diagnosis. 76.5% (88/115) of patients with febrile UTI had elevated CRP vs. 25.0% (7/28) in the group without fever.

The average value of blood leukocytes was 15,050/mm^3^ (p25-75: 10,750 to 20,100) with a minimum of 3,800/mm^3^ and maximum of 39,000/mm^3^. The value of neutrophils in the blood had a median of 62% (p25. -75: 47-78), and ranged from a minimum of 6% ​​to a maximum of 91%.

Leukocyturia was found in 72% of patients, leukocyte esteareasas in 69.4% of patients, positive for nitrites in 34.5%, hematuria in 34% and proteinuria in 61.1%. In all cases, gram staining of a drop of urine uncentrifuged was conducted and positives were obtained in 86.0% of the samples, with 77.8% of bacilli were gram stain negative.

More than sixty-nine percent (69.4%) of urine cultures were taken by an evacuating catheter and the rest came from spontaneous voiding. Urine cultures were taken by catheterization in 75% of children under three years and in 34% of those over four years.

The most common uropathogens were bacillus with gram staining negative; mainly *E.coli* and *Klebsiella pneumoniae* (see Table 3). The taking of blood cultures was performed on 22.9% (33 patients), of which only three were positive (9.1%).

**Table 3.**
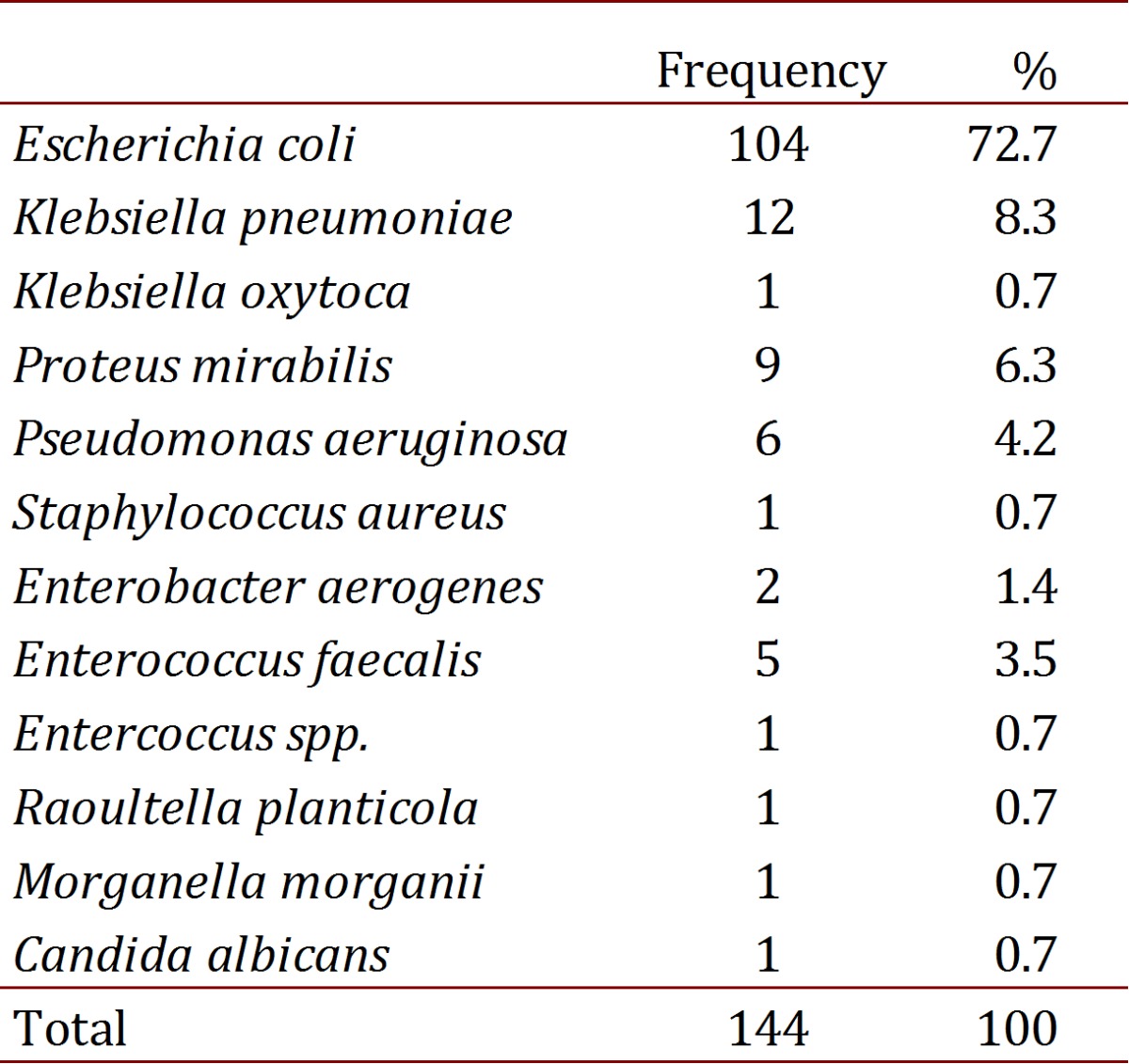
Isolation on the urine culture

### Resistance profiles:

Table 4 reports on resistance profiles in this population group. *Escherichia coli* had a resistance of less than 10.0% to amikacin, cefepime, ceftazidime, meropenem, ciprofloxacin, and piperacillin tazobactam, and of 18.0% to nitrofurantoin and had a resistance of 43.0% and 47.6% to trimethoprim-sulfamethoxazole and ampicillin sulbactam, respectively. The resistance profile to other antibiotics was: gentamicin (12.0%) and nalidixic acid (21.4%). The percentage of *Escherichia coli* in the presence of extended spectrum beta lactamase (ESBL) was 6.3%, and 15.4% for *K. pneumoniae*. Only five episodes were observed of UTI by *Enterococcus faecalis*, none of these were resistant to vancomycin.

In total, 16 patients had previously received an antibiotic prophylaxis. In these patients the percentage of *E. coli* resistance to ampicillin-sulbactam was 60.0% as compared to 45.3% in those who had not previously received a prophylaxis (*p*= 0.659). Of the patients who had previously presented with UTI, 36.4% were resistant to ampicillin sulbactam vs. 43.6% of patients with no history of previous UTI (*p*= 0.499).

**Table 4.**
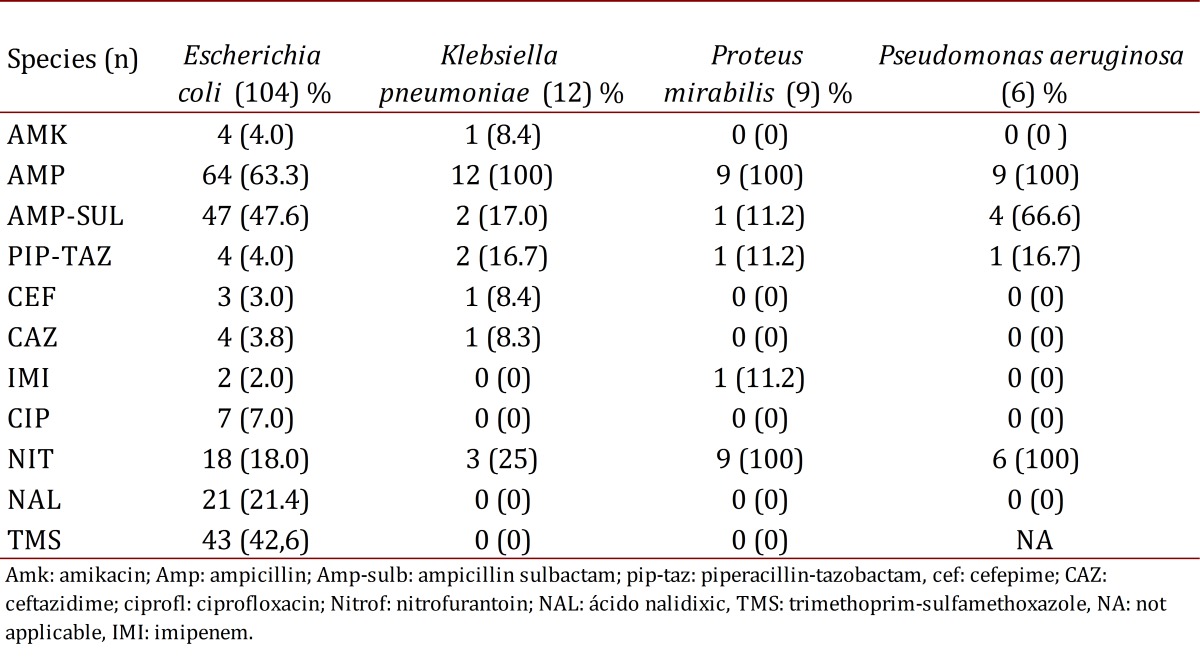
Isolating antibiotic resistance

### Treatment:

Amikacin was used for 66.0% of patients (95), followed by ampicillin sulbactam in 9.7% (14), ciprofloxacin in 11.8% (17), piperacillin-tazobactam in 4.2% (6), nalidixic acid 2.1% (5) and cefazolin in 1.4% (2). 92.4% (133) received intravenous treatment and of these, 32.6% (47) were changed to oral medication. The timing of the change to oral medication had a median of 4 days (p25-75: 3-5), the total duration of treatment received was 7 days (p25-75: 7-10), with a range of 5-28 days. All patients had an adequate response to the treatment conducted. In 89.7% of patients the fever disappeared in less than 48 hours. Two patients had nephronia as a complication associated with the UTI. There were no deaths.

### Findings from radiological imagery

Ultrasound was performed for 127 patients and some abnormality was found in 39 patients (30.7%). Voiding cystourethrogram (VCUG) was performed on 97 patients (67.4%), 79 were normal and 18 patients had some degree of VUR, which is equivalent to 18.5% of the study population with VCUG.

## Discussion

The appropriate antibiotic choice for UTI requires adequate understanding of the epidemiology and the profile of local antimicrobial resistance of associated uropathogens which is not applicable to all geographical regions. This study describes the resistance profile of uropathogens causing UTI in a pediatric population of a hospital in the city of Medellin, Colombia. The majority of UTI episodes were caused by Gram-negative bacteria, mainly *E. coli* (72%). This is consistent with the microbiological profile of patients with acquired UTI in the community that has been reported in articles published in recent years in different regions of the world 4 ^,^ 10.

The choice of antibiotics in UTI depends on the local resistance pattern. Drugs such as cephalosporins, sulfamethoxazole trimethoprim and amoxicillin-clavulanate are the most utilized oral antibiotics 11 ^,^ 12. For intravenous treatment there is ampicillin-sulbactam, cephalosporins of second and third generation and aminoglycosides. Resistance of enterobacteriaceae to beta*-l*actam antibiotics has increased in the past 30 years 11. This was first detected in Germany in 1980 and was quickly reported in the United States 13. This resistance is principally observed in *E. coli* and *Klebsiella*, but can also be identified in other enterobacteria.

The results obtained from this study showed a high resistance of enterobacteria to ampiclina sulbactam and trimethoprim sulfamethoxazole. This is similar to recent articles in which several authors report resistances that fluctuate between 24 and 58% for ampicillin sulbactam and are up to 36% for trimethoprim sulfametoxazol 10 ^,^ 14 ^,^ 15. In our hospital the high resistance of *E. coli* to ampicillin sulbactam (47.6%) was a disturbing finding since this is one of the most widely used empirical therapies for febrile UTI. Amikacin, however, showed a resistance of 4% for *E. coli* and less than 10% for *K. pneumoniae* and *Pseudomonas aeruginosa*.

Empirical therapy also depends on the severity of the UTI. For example, the patient with complicated UTI, pyelonephritis, or uro sepsis 10 should receive intravenous antibiotic treatment. In these cases, cephalosporins of the third and fourth generation could be an option as their resistance profile was low. Ceftazidime and cefepime reported *E. coli* resistance as less than 4%, similar to previous studies 11 studies; however, the risk of inducing the production of ESBL should be present, which were reported in 6.3% of *E. coli* infections in this study.

Nitrofurantoin is a bacteriostatic agent for gram positive but a bactericide for gram negative, which has become an oral alternative for urinary infections 11 ^,^ 15 ^,^ 16. Some studies have shown that nitrofurantoin has an adequate renal excretion and is recommended for complicated urinary tract infections 17. In this study little resistance was found to nitrofurantoin, but for being only a bacteriostatic antibiotic it is not recommended in complicated urinary tract infections. Other medications that showed low resistance (7%) was ciprofloxacin for reported uropathogens.

At present it is not clear what is the most effective therapy and appropriate antibiotic treatment time for UTI, but a review of the Cochrane collaboration of 2012 found that 10 days of antibiotic treatment was effective in eliminating bacteriuria 1. In our study, the average treatment time was 7 days (p25-75: 7-10) with an adequate clinical response to the treatment used.

Although it was not intended in this study to carry out an association between symptoms at the time of UTI diagnosis, a relevant finding was that 37.1% of children under two years of age had associated respiratory symptoms. It is noteworthy that some studies have shown that the presence of respiratory symptoms does not exclude the possibility of presenting with UTI 5. Kupperman et al. found a prevalence rate of 4.1% prevalence of UTI in patients with bronquiolitis 18 and Levine et al. documented UTI rate of 5.4% in patients with respiratory infection by respiratory syncytial virus 19. These findings suggest the need to search for UTI in pediatric patients under two years of age with respiratory symptoms.

Of patients having a voiding cystourethrogram (97/144), a significant frequency of VUR was found (18.5% of those having a cystourethrogram performed, 12.0% of all patients), although no conclusions can be drawn with this population as they are selected patients from a hospital of high complexity. This data reinforces the findings previously found for the group. For this reason, it is suggested that UTI in a pediatric population is a marker of malformations of the urinary tract as it is found in up to 78.3% of patients diagnosed with febrile UTI 20 and that may promote the development of renal scarring with the long-term risk of hypertension, proteinuria and chronic renal failure 21. These data highlight the importance of evaluation and imaging follow-up in all pediatric patients diagnosed with urinary tract infection.

## Conclusion

Empirical treatment with sulfamethoxazole and trimethoprim ampiclina-sulbactam for community-acquired UTIs may be insufficient due to the elevated rate of resistance of *E. coli* and other isolated uropathogens in our institution. Conversely, amikacin, ciprofloxacin and nitrofurantoin would be therapeutic alternatives in pediatric patients with uncomplicated UTI and cephalosporins of the 3rd or 4th generation in those with more severity, as in the case of urosepsis.
